# Salivary Morning Cortisol as a Potential Predictor for High Academic Stress Level in Dental Students: A Preliminary Study

**DOI:** 10.3390/ijerph19053132

**Published:** 2022-03-07

**Authors:** Kacper Nijakowski, Dawid Gruszczyński, Kacper Łaganowski, Jagoda Furmańczak, Alicja Brożek, Marcin Nowicki, Dorota Formanowicz, Anna Surdacka

**Affiliations:** 1 Department of Conservative Dentistry and Endodontics, Poznan University of Medical Sciences, 60-812 Poznan, Poland; annasurd@ump.edu.pl; 2 Student’s Scientific Group in Department of Conservative Dentistry and Endodontics, Poznan University of Medical Sciences, 60-812 Poznan, Poland; dawid.j.gruszczynski@gmail.com (D.G.); kacperloo111@gmail.com (K.Ł.); jagodafurmanczak@gmail.com (J.F.); 3 Department of Medical Chemistry and Laboratory Medicine, Poznan University of Medical Sciences, 60-806 Poznan, Poland; abrozek@ump.edu.pl (A.B.); nowickim@ump.edu.pl (M.N.); doforman@ump.edu.pl (D.F.)

**Keywords:** cortisol, total antioxidant status, saliva, stress, dental students

## Abstract

Students experience different levels of acute and chronic stress during the academic year. Selected salivary biochemical parameters change as a result of stress. Our preliminary study aimed to indicate possible links between alterations in the salivary biochemical parameters (such as cortisol and total antioxidant status) and different accompanying stress levels in dental students during the academic year. The study group consisted of 20 volunteer dental students at the Poznan University of Medical Sciences—both genders, aged 20–26 years. Students were asked to fill in the electronic version of the author’s survey on experiencing and coping with stress. Samples of unstimulated saliva were collected in the morning and late evening at four-time points: in the middle of the academic year, during the examination period, at the beginning of the academic year, and in the middle of the following academic year, together with a determination of currently experienced stress on the Stress Numerical Rating Scale-11. According to the circadian rhythm of cortisol secretion, morning levels of the hormone in saliva were much higher than in the evening. In evening cortisol, significant differences were observed during the studied periods—the highest level was found at the beginning of the academic year. However, the morning cortisol concentrations correlated more strongly with the declared stress levels and showed better predictability for high-stress levels. Salivary morning cortisol could be a potential marker of academic stress levels. Further studies are needed on a larger group to confirm.

## 1. Introduction

Stress is regarded as the response of the body to any physical or psychological challenges that disrupt homeostasis. This is expressed by several cognitive, emotional, and behavioural changes to address this challenge [1,2]. Whereas short-term stress can boost the immune system, long-term stress is deleterious to immune function and often leads to numerous physical and mental disorders, including infectious illnesses, diabetes, cardiovascular disease, and certain cancers [3,4]. Academic stress has been shown to manifest as anxiety, depression, emotional exhaustion, fatigue, gastrointestinal symptoms, and sleeplessness. Additionally, an adverse effect of stress on educational achievement has been observed [5]. Dental education is considered to be highly demanding academically and clinically [6,7]. Therefore, it is linked with a high prevalence of psychological stress among students. Interestingly, dental students present higher levels of stress than the general population [8,9]. Dentistry stress includes living, personal, educational, academic, and clinical factors [5].

It has been reported that stress differs according to the year of study. Most previous studies demonstrated a higher stress level among students participating in clinical classes [5]. However, there is no consensus on which moment of academic education is associated with the highest level of psychological stress. Some studies indicate a period of transition from preclinical to clinical activities due to concerns in first interpersonal contact with patients and the performance of required treatment procedures [8,10,11]. In contrast, other researchers observed that the most stressful aspect is to improve practical skills in the final year of studies, which is undoubtedly connected with anxiety concerning future professional work [5,9]. Moreover, during the academic year, students may experience different stress levels, e.g., increasing in the examination sessions. The literature reports that changes in selected salivary biochemical parameters may allow non-invasive and stress-free monitoring of stress levels and thus prevent the development of mental health disturbances [12].

Widely known as the stress hormone, cortisol is released from the adrenal cortex in response to stress. The measurement of cortisol concentration in saliva has several advantages over the more conventional and commonly used total serum concentrations. Obtaining saliva samples for cortisol determination is simple, non-invasive, and stress-free, whereas blood sampling may be stressful and thus elevate cortisol levels [13]. Salivary cortisol levels are independent of saliva flow rate and correlate with the biologically active, unbound fraction of plasma and serum cortisol [14,15]. Furthermore, the time lag between alterations of cortisol levels in plasma and saliva is 1 to 2 min [16]. An additional advantage is that cortisol is stable in saliva for up to three months at 5 °C and at least one year at −20 °C or −80 °C. However, long-term storage of saliva samples at room temperature is not recommended because of decreased cortisol concentrations every month.

Furthermore, repeated cycles of freezing and thawing have no impact on cortisol levels [17]. On the other hand, snack eating affects salivary cortisol; therefore, no food should be consumed for at least 90 min prior to saliva sampling [18]. Moreover, there is a potential impact of drugs (e.g., glucocorticoids, antidepressants) and diseases (e.g., hypothalamic-pituitary-adrenal axis disorders) [19,20]. Additionally, salivary cortisol differs with age and sex. Salivary cortisol levels have been observed to increase in response to stress in older people, especially males [21].

Free radicals, both reactive oxygen species (ROS) and reactive nitrogen species (RNS), are the products of normal cellular metabolism. In low or moderate concentrations, they play an essential role in various cell processes, but it seems that they are primarily signalling molecules. However, higher levels of free radicals can cause damage to macromolecules, such as lipids, proteins, and nucleotides, resulting in impaired cell function and death [22]. Increased ROS production may be caused by external factors such as ultraviolet radiation, ionising radiation, pollution, pesticides, toxic metals, unhealthy lifestyle, and long-term stressful conditions [23,24]. Among the possible sources of ROS in the oral cavity can be mentioned periodontal diseases, xenobiotics (e.g., tobacco, ethanol), high-fat or high-protein diet, and dental materials (e.g., fluorides, resin composites containing methacrylates) [25]. Under physiological conditions, their production is controlled by the antioxidant defence system (ADS). Disruption of the balance between the intensity of oxidative processes generating ROS and their elimination by ADS is defined as oxidative stress [26]. In contrast, total antioxidant capacity (TAC) reflects all antioxidants’ cumulative response in biological fluids [27,28]. Numerous studies have argued that oxidative stress is involved in the pathogenesis of multiple disorders, including cancer, cardiovascular diseases (atherosclerosis), diabetes mellitus, neurodegenerative diseases (Alzheimer’s disease, Parkinson’s disease, and multiple sclerosis), rheumatoid arthritis, respiratory diseases (asthma), as well as, the ageing process [22,29,30].

Our preliminary study aimed to indicate possible links between alterations in the selected biochemical parameters of saliva (such as cortisol and total antioxidant status) and different accompanying stress levels in dental students during the academic year.

## 2. Materials and Methods

### 2.1. Study Participants

The study group consisted of 20 healthy volunteers dental 3rd- and 4th-year students at the Poznan University of Medical Sciences—both genders (14 females), aged 20–26 years. The exclusion criteria were: autoimmune diseases (including diabetes), endocrine disorders concerning adrenal gland hormone secretion, obesity, mental disorders (including depressive and anxiety disorders, smoking, periodontal disease, active caries, pregnancy, high-fat or high-protein diet, regular antioxidant supplementation, corticosteroids, and drugs affecting saliva secretion. The medical history data was obtained based on anamnesis. All students had a clinical dental examination to exclude the influence of oral inflammation. The participants demonstrated perfect oral hygiene without any signs of even localised gingivitis. Students were asked to fill in an electronic version of our survey on experiencing and coping with stress.

### 2.2. Saliva Samples

The material for laboratory tests was unstimulated mixed saliva. The saliva was always collected at the same time of day for 10 min. All subjects had to be examined at least 2 h after the meal. Each student should be in a relaxed sitting position, with the trunk slightly inclined towards the knees, so that the saliva can flow freely into the collection container. During the procedure, students were asked not to swallow their saliva. The samples rested in an ice container from the start of the saliva collection until it was centrifuged.

Samples of saliva were collected from students in the morning (between 6 a.m. and 7 a.m.) and late evening (between 10 p.m. and 11 p.m.) at four time points: in the middle of the academic year (January/February 2019), during the examination period (mid-June), at the beginning of the academic year (first half of October) and in the middle of the following academic year (January/February 2020), together with a determination of currently experienced stress using the Stress Numerical Rating Scale-11 (Stress NRS-11 with rates between 0 and 10).

Biochemical analysis of the saliva was performed in the Department of Clinical Biochemistry and Laboratory Medicine at the PUMS. Saliva samples were centrifuged for 15 min at 3000 rpm using Eppendorf Centrifuge 5702, separated into Eppendorf tubes, and frozen at −80 °C until laboratory determination. Concentrations of selected markers—cortisol and total antioxidant status—in the saliva were determined using the appropriate diagnostic kits (respectively, Salivary Cortisol—DRG Diagnostics and Human Total antioxidant capacity—SunRed). TAS was evaluated only for the morning salivary samples.

### 2.3. Statistical Analysis

The Shapiro–Wilk test indicated no normal distribution of continuous variables. Due to repeated measurements, the assessed levels were compared with the non-parametric ANOVA Friedmann test. The Spearman correlations were performed. The predictive value of selected markers was evaluated in the logistic regression and the ROC analysis using all 80 salivary samples. Data were analysed using Statistica 13.3 software (StatSoft, Cracow, Poland). For all analyses the significance level was set at α = 0.05.

## 3. Results

### 3.1. Sociopsychological Factors

According to the survey, among the most common causes of stress in everyday life, 80% of the respondents reported studies, and 20%—family matters or social life. More than half of the students declared the stress related to the excess of learning, and nearly half of them—the stress present during exams, especially oral ones. The most common symptoms of the stress experienced were nervousness (85%), palpitations (45%), hand tremors (35%), headaches (30%), excessive sweating (30%), gastrointestinal problems (30%), or xerostomia (25%). The respondents most often coped with stress by talking to their family or friends through entertainment (TV, Internet) and snacking—respectively, 75%, 80%, and 50%. Stress motivated 50% of students, paralyzed 35%, and was irrelevant to 15%.

**Figure 1:**
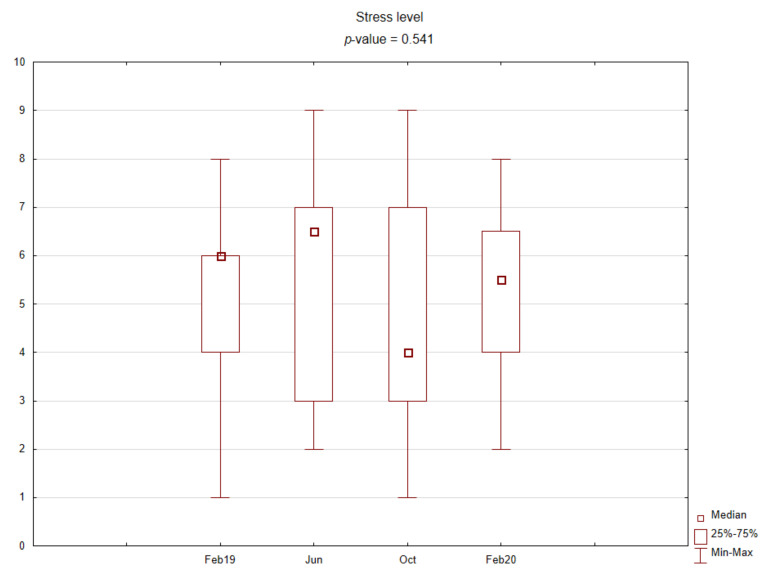
Box plot for experienced stress levels using Stress NRS-11 depending on time point.

Figure 1 shows perceived stress levels using Stress NRS-11 depending on the time point of the study.

### 3.2. Biochemical Analysis

**Figure 2:**
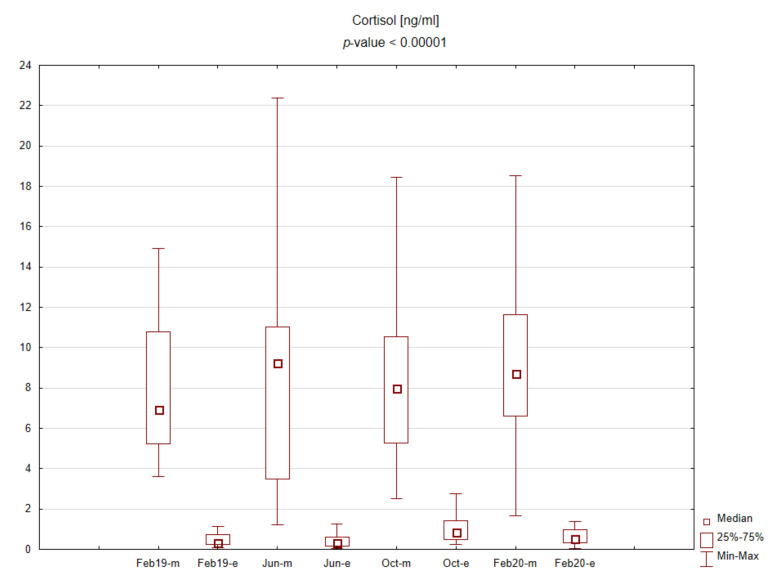
Box plot for cortisol concentrations in saliva depending on time point.

**Figure 3:**
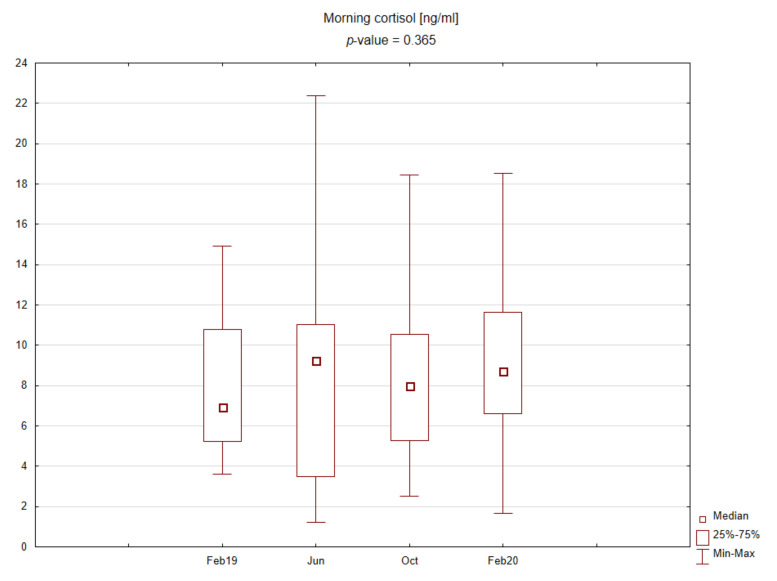
Box plot for morning cortisol concentrations in saliva depending on time point.

**Figure 4:**
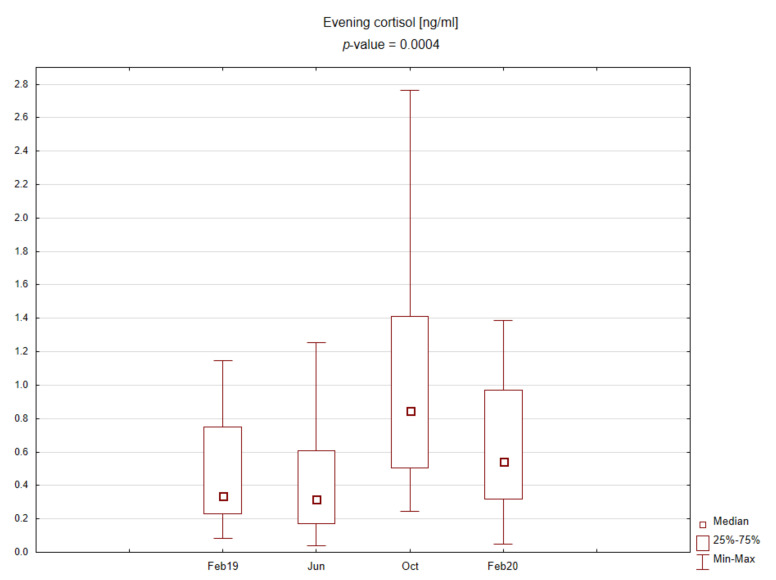
Box plot for evening cortisol concentrations in saliva depending on time point.

**Figure 5:**
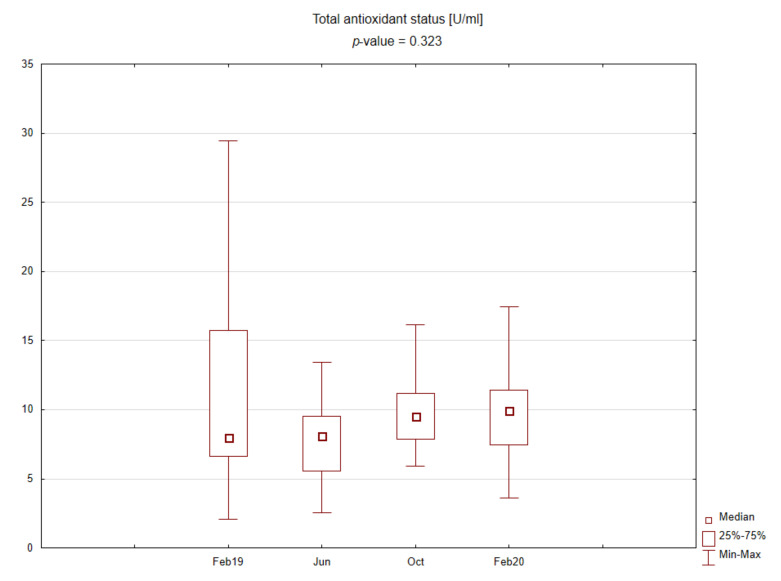
Box plot for salivary total antioxidant status depending on time point.

The central part of the study was laboratory analysis. The results are presented graphically on the box plots (Figure 2, Figure 3, Figure 4 and Figure 5). Due to the time of day and year variability in saliva secretion, no detailed data have been reported. However, no statistically significant stress-related differences in saliva flow rate were found.

Evening salivary cortisol levels were significantly lower than morning levels. Morning cortisol concentrations were slightly higher during the exam period, and, interestingly, evening cortisol concentrations were significantly elevated upon returning after the summer holidays. No significant differences were found for the TAS level during the academic year.

Moreover, there was no clear association between the examined factors, regardless of the time point. The morning cortisol concentration increased with growing stress levels, which was not observed for evening cortisol. The TAS did not show any specific trend. Values of Spearman correlation coefficients are included in Table 1, Table 2, Table 3 and Table 4.

**Figure 6:**
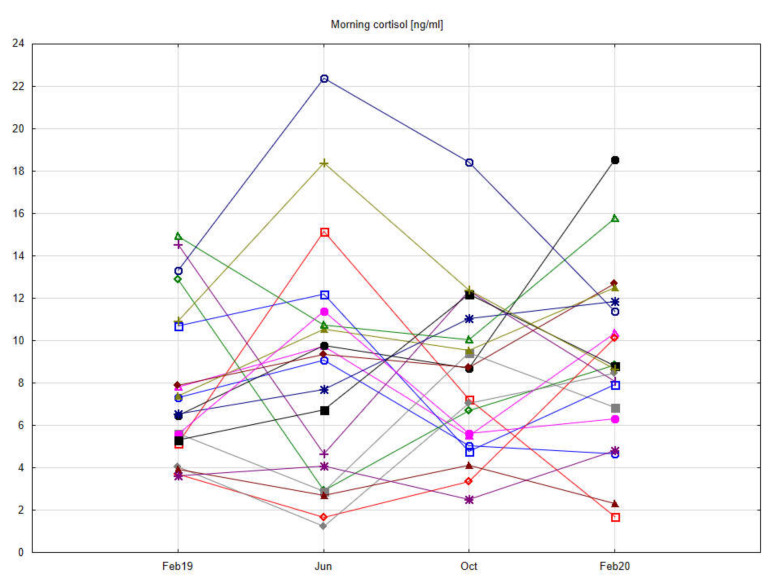
Profile graph showing changes in morning salivary cortisol in individual students.

Figure 6 shows individual changes in salivary morning cortisol, suggesting a tendency to detect higher levels of academic stress in students.

Model of logistic regression was constructed with the forward stepwise approach—parameters were presented in Table 5. In the presented logistic model, only morning cortisol was a statistically significant predictor included in the model. With an increase in salivary cortisol by one unit, the odds of being in a high-stress group (for values ≥ 7 in the survey) increased by 18%.

**Figure 7:**
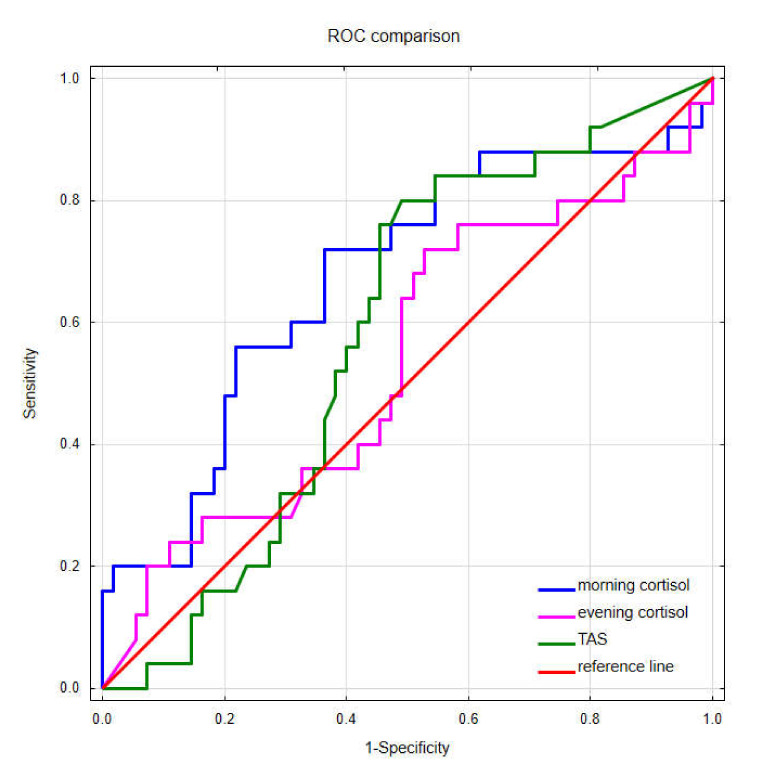
Comparison of receiver operating characteristic curves for morning cortisol, evening cortisol, and total antioxidant status in saliva.

Moreover, Figure 7 presents the ROC curves performed for predictive assessment of high-stress levels (for values ≥ 7 in the survey) by the determined salivary markers. Only the curve for morning cortisol concentration differs significantly from the reference line (AUC = 0.673, SE = 0.068, *p*-value = 0.011). The AUC for evening cortisol and TAS are, respectively, 0.540 ± 0.072 and 0.584 ± 0.065.

The above results suggest that morning cortisol could be a potential marker of stress levels in saliva.

## 4. Discussion

In our study, according to the diurnal rhythm of cortisol secretion, morning salivary concentrations of the hormone were significantly higher than evening ones. Significant differences were observed only for evening cortisol across the study periods, with the highest levels found at the beginning of the academic year. In contrast, morning cortisol concentrations correlated more strongly with reported stress levels and showed better predictive power for high-stress levels (above six on the questionnaire).

A similar study to ours was conducted by Batabyal et al. [31] on a group of first-year students in India. The study group was of similar size, and saliva samples were collected only in the morning at six-time points during one academic year. Stress levels were determined using psychological scales, such as PSS14 (Perceived Stress Scale) and K10 (Distress Scale). Males had significantly lower cortisol levels compared to females and a tendency for experienced stress to decrease the closer the academic year ended. In contrast, no significant differences in stress levels were observed in females during the academic year. In this subgroup, only an increase in cortisol levels was found midway through the second semester relative to the beginning of the academic year. By both genders, academic stress was declared as the most common source of distress across all time points of this longitudinal survey.

In another study, Pani et al. [32] assessed salivary cortisol levels in final-year Saudi dental students at three-time points: the beginning of the semester, the last week of clinical classes, and one hour before the final examination. Cortisol concentrations immediately before the exam were significantly highest, whereas initial concentrations were significantly lowest relative to the other periods. When dividing participants by gender, significantly higher cortisol levels were found for males. Interestingly, students with a very high GPA (grade point average) had lower cortisol levels in stimulated saliva, especially during the exam. Cortisol concentrations in this period negatively correlated with perceived academic stress. Students who were worried about their grades were relatively more relaxed immediately before the examination than the rest.

More studies have focused on comparing salivary levels of stress markers during exam periods and relatively stress-free periods in the literature. Irshad et al. [12] found significant psychological changes in students before the exam (increased anxiety and life stress and decreased well-being), reflected in biochemical changes in saliva. Salivary morning cortisol levels increased significantly in participants during the examination time. Additionally, the secretion of free light chains significantly decreased, and immunoglobulin A showed a clear downward trend. In the study by Murphy et al. [33], elevated perceived acute exam stress was associated with significantly elevated salivary evening cortisol concentrations. Immunoglobulin A levels decreased but not significantly. Students who spent more hours studying during the examination week had lower cortisol levels in saliva (significant negative correlation between these variables).

Moreover, Ng et al. [34] determined differences in experienced stress and levels of salivary markers (such as cortisol, IgA, and chromogranin A) immediately before and after the exam test. Before the test, students reported higher levels of perceived stress, which resulted in a significant increase in cortisol and a slight decrease in the secretion of IgA and chromogranin A. Stress scores correlated inversely with exam scores—the relationship was stronger for pre-test levels. Similar results showing the association of increased exam stress with elevated salivary morning cortisol levels were obtained by other authors [35,36]. In contrast, several studies have found no significant association between changes in stress or anxiety with increases in salivary cortisol levels during the examination session [37,38].

However, few studies address the relationships of psychological stress with salivary oxidative stress in students. Piedade Sequeira and Naik [39] observed significantly lower salivary total antioxidant capacity (TAC) on exam day than in the post-exam period. Furthermore, a strong inverse correlation was found between stress levels and TAC of saliva. The authors suggest salivary TAC as a potential effective marker of psychological stress and recommend considering antioxidant supplementation in students during exam periods associated with increased stress levels.

In addition to the exam period, high-fidelity simulation (HFS) can also be stressful for students. Bialka et al. [40] evaluated the effect of HFS on stress levels in final-year medical students during critical care classes. At three time points: before starting the scenario, after the end of the scenario and 120 min after that, they measured heart rate (HR), systolic blood pressure (SBP), diastolic blood pressure (DBP), mean blood pressure (MBP), oxygen saturation (SpO2), and collected saliva samples to determine alpha-amylase activity and IgA, cortisol, and testosterone concentrations. Among hemodynamic parameters, SBP, MBP, and HR were significantly higher immediately after the end of the scenario. Similarly, alpha-amylase activity was highest at this time point.

In contrast, IgA levels were higher before starting the scenario and cortisol levels 2 h after the scenario, but these differences were not statistically significant. The only variable that showed a significant difference was testosterone level which was higher at the last measurement point. Interestingly, team leaders showed significantly lower cortisol and alpha-amylase concentrations. The authors assume that HFS can induce stress levels comparable to future clinical work based on these findings. Other factors that elevate stress levels include occasional shift work [41]. These students demonstrated significant changes in the biochemical parameters of saliva—the increase of morning cortisol concentration and the decrease of the antioxidant activity.

The literature also includes studies on ways to reduce exam stress levels and their effects on the salivary biochemical profile of students. Pani et al. [42] evaluated the effects of regular physical activity on psychological stress levels and salivary TAC. Although there were no significant differences in levels of reported exam stress, a smaller decrease in TAC was observed in the physically active group. These findings suggest that regular exercise may protect students from oxidative stress associated with academic stress. Furthermore, Gebhart et al. [43] assessed the effectiveness of methods such as animal-assisted therapy with dogs, music therapy with body percussion, and mandala painting. Similarly, the intervention and no-intervention groups did not significantly differ in self-reported perceived stress levels. However, students with distraction-focused interventions had significantly lower cortisol levels and increased secretory immunoglobulin A levels during the non-stress and exam periods compared to controls. Interestingly, Vrbanović et al. [44] also observed a decrease in psychological stress in female patients with temporomandibular disorders after a 3-month splint therapy, which positively correlated with salivary morning cortisol levels.

Psychological stress can also be reflected in changes in markers measured in the blood samples. Al Qteishat et al. [2] observed that students under exam stress present significantly higher cortisol and adrenaline levels and intensification of lipid peroxidation one hour before and one hour after the exam compared to the control group. Malonic aldehyde, isolated double bonds, diene conjugates, ketodienes and conjugated trienes, reduced glutathione, and catalase were determined among oxidative stress markers. Although the baseline alterations decreased with time after the exam, they still remained higher than the control group after one day. Moreover, glucose levels were higher in response to exam stress due to hypercortisolaemia. Despite the determination of different parameters, similar changes in antioxidant status were observed in other studies concerning the stress level in students [45,46,47]. 

Continued chronic stress is associated with an increased risk of burnout syndrome, especially in medical professionals because of the specific work environment. Deneva et al. [48] showed that physicians with burnout syndrome had significantly elevated serum and salivary cortisol levels. Additionally, salivary cortisol concentrations were significantly correlated with job burnout in a logistic regression model.

Limitations of our preliminary study may include a relatively small sample size; however, saliva samples were collected at several time points, which increased the number of measurements. In addition, the level of perceived academic stress was rated by students subjectively. It was impossible to rule out the interference of the stress level by other various stressors in private life. Among the advantages, saliva collection as diagnostic material is stress-free in contrast to serum. Moreover, possible sources of oxidative stress in the oral cavity were excluded by the dental examination.

## 5. Conclusions

Dental students experience different levels of stress during the academic year. Morning cortisol in saliva could be a potential marker of perceived stress in these students. Further studies with a larger group are needed for confirmation.

## Figures and Tables

**Table 1:** Spearman correlation coefficients—February 2019.

	Morning Cortisol	Evening Cortisol	TAS
stress level	0.311	0.297	0.232
morning cortisol		0.414	0.694 *
evening cortisol			0.193

* Statistical significance *p*-value < 0.05.

**Table 2:** Spearman correlation coefficients—June.

	Morning Cortisol	Evening Cortisol	TAS
stress level	0.348	0.001	−0.256
morning cortisol		0.301	−0.089
evening cortisol			0.137

**Table 3:** Spearman correlation coefficients—October.

	Morning Cortisol	Evening Cortisol	TAS
stress level	0.345	−0.248	−0.119
morning cortisol		−0.073	0.124
evening cortisol			0.137

**Table 4:** Spearman correlation coefficients—February 2020.

	Morning Cortisol	Evening Cortisol	TAS
stress level	0.309	−0.027	0.090
morning cortisol		0.095	0.263
evening cortisol			0.075

**Table 5:** Parameters of predictors incorporated into the logistic regression model.

	β	SE	Wald Stat.	*p*-Value	Odds Ratio	Confidence OR −95%	Confidence OR 95%
intercept	−2.241	0.626	12.794	<0.001 *	0.106	0.031	0.363
morning cortisol	0.163	0.062	6.925	0.009 *	1.177	1.043	1.330

* Statistical significance *p*-value < 0.05.

## Data Availability

Data are available on request from the corresponding author.
